# From Nigeria to innovation: A vision for inclusive science

**DOI:** 10.1016/j.isci.2025.111780

**Published:** 2025-02-06

## Abstract

RBSA honorable mention.

## Main text

**Figure:**
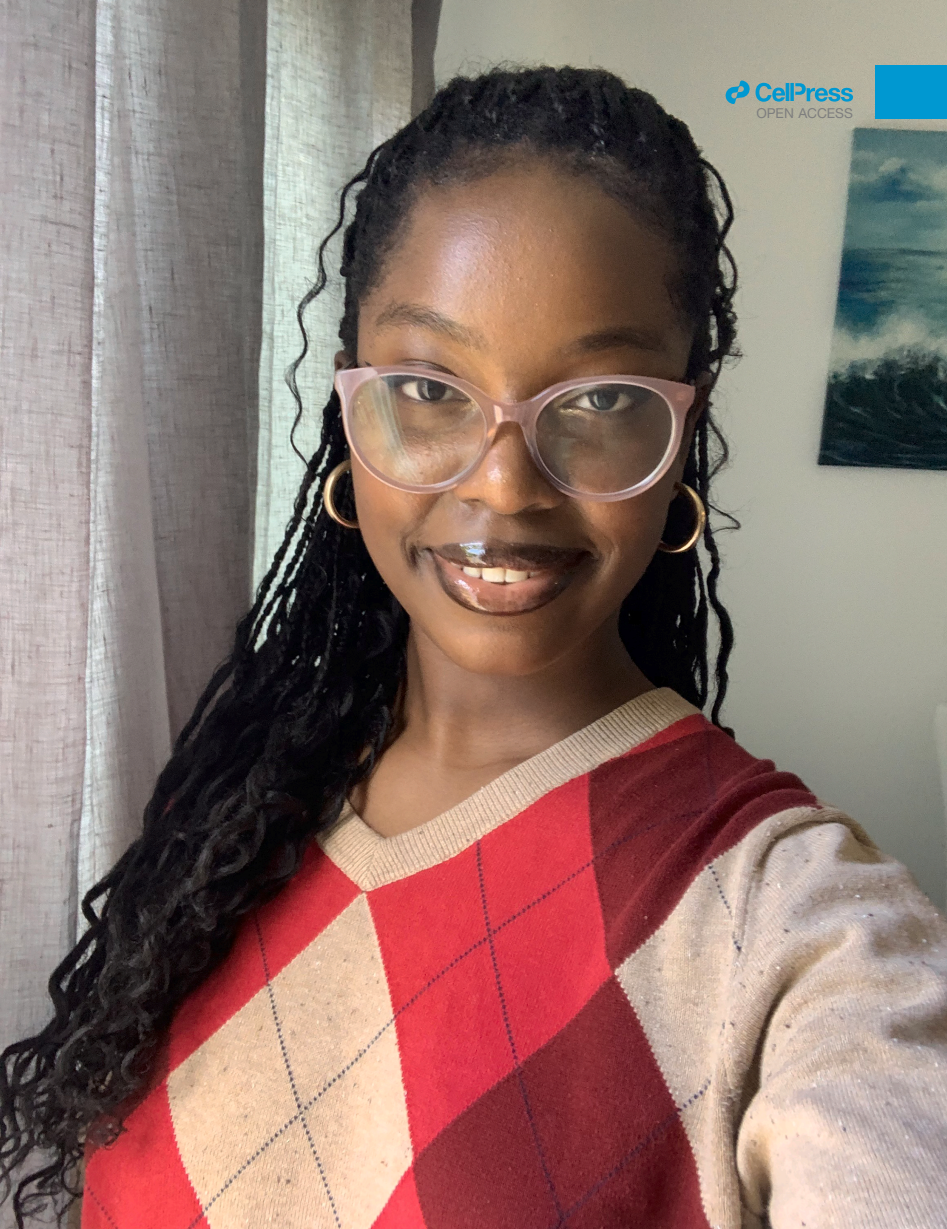
Above image: Efemena Johnson, author of From Nigeria to innovation: A vision for inclusive science

Growing up in Nigeria, I was surrounded by vibrant cultures and communities, but I was also acutely aware of the gaps in access to healthcare and technology. In many rural areas, preventable diseases claimed lives because of a lack of medical resources. Seeing this firsthand sparked my interest in science, particularly in how technology could bridge these gaps. I was fascinated by the possibility that a single innovation—whether a diagnostic tool or a mobile health app—could save lives. This experience ignited my passion for creating solutions that have a direct impact on underserved communities.

The turning point in my scientific journey came during my research assistantship at Marymount University, where I conducted soil sampling to isolate antibiotic-resistant bacteria. I remember the moment I first saw the resistant strains under the microscope. It struck me that the bacteria evolving in those samples mirrored the real-world struggle in places like Nigeria, where antibiotic resistance has severe consequences due to limited healthcare infrastructure. This project made me realize that science could do more than provide answers—it could offer solutions to critical global health challenges. It was this realization that motivated me to pursue an interdisciplinary approach, blending biochemistry with computer science to tackle these issues from multiple angles.

My transition to George Mason University to study computer science gave me a new platform to apply my skills, particularly in the realm of machine learning. One of my most fulfilling projects has been the development of a job search platform for individuals with autism. Having grown up in a community where access to specialized resources was often lacking, I understood the importance of designing tools that cater to diverse needs. By analyzing user data and creating adaptive models, I aimed to make the job search process more inclusive. The moment I saw the platform’s first successful matches, I felt a deep sense of fulfillment; knowing that my work could help neurodiverse individuals secure employment. This experience reinforced my belief that technology should not only drive innovation but also champion inclusivity.

Working on predictive analytics for healthcare further deepened my passion for applying machine learning to solve real-world problems. I vividly remember the first time our model successfully predicted hospital readmissions using patient data from public healthcare datasets. It was a powerful moment. Coming from Nigeria, where access to advanced healthcare tools is often limited, I saw how machine learning could revolutionize healthcare by making early interventions possible in under-resourced areas. This realization underscored my commitment to using data science to make healthcare not only smarter but also more accessible to communities around the world.

Climate change analysis is another area where my background in Nigeria has deeply shaped my perspective. Growing up, I witnessed the impact of environmental degradation and deforestation, particularly in rural communities. Working on climate forecasting projects at George Mason, where I used techniques like ARIMA and LSTM networks to project future scenarios, felt intensely personal. The knowledge that I could contribute to research that might protect vulnerable communities from the devastating effects of climate change reaffirmed my desire to be part of a scientific community that serves the greater good.

At the heart of my scientific vision is the belief that science should serve everyone, especially those from underserved communities. My interdisciplinary background in biochemistry and computer science gives me a unique perspective on solving problems, allowing me to break down traditional barriers between fields. I am committed to ensuring that the tools and solutions we develop are accessible to all, regardless of their background. This belief in inclusivity is not only refiected in my work on neurodiverse user interfaces but also in my broader approach to science. I aim to create solutions that can be scaled and adapted to meet the needs of diverse populations, especially in developing regions.

Community has always been a vital part of my scientific journey. Programs like the Qualcomm Student Accelerator and Amazon’s Reach Back DC have been instrumental in shaping my understanding of mentorship and support networks. At Qualcomm, I had the opportunity to collaborate with peers who shared my vision for an inclusive future, while at Amazon’s Reach Back DC, I met mentors who inspired me to pay it forward.

These experiences taught me the importance of fostering a scientific community that supports underrepresented students, just as I have been supported. I am committed to mentoring others, particularly students from marginalized backgrounds, to ensure that they have the resources and encouragement they need to pursue their own scientific dreams.

Looking forward, I see my career continuing at the intersection of biotechnology and data science. I am particularly interested in leading interdisciplinary teams that address global challenges such as antibiotic resistance, healthcare access, and climate change. I envision applying machine learning to accelerate research in these areas, while also ensuring that the solutions we create are inclusive and accessible. For instance, I plan to develop AI-driven tools that can be deployed in rural areas to provide early disease detection and intervention, something I wish I had seen more of in my community growing up in Nigeria.

Growing up in Nigeria instilled in me a deep sense of responsibility to use science as a tool for social good. I have seen the effects of inequality in healthcare and technology access, and I am determined to close those gaps. As I continue my journey, I am excited to contribute to a future where science empowers all people, regardless of where they come from, and where innovation is used to create a more equitable world. My vision is to lead not just through innovation, but through inclusion, ensuring that the benefits of scientific progress reach everyone.

